# Editorial for the Genetics of Muscular Dystrophies from the Pathogenesis to Gene Therapy Special Issue

**DOI:** 10.3390/genes14040926

**Published:** 2023-04-17

**Authors:** Luisa Politano, Filippo M. Santorelli

**Affiliations:** 1 Cardiomyology and Medical Genetics, Department of Experimental Medicine, University of Campania “Luigi Vanvitelli”, 80138 Napoli, Italy; 2 IRCCS Fondazione Stella Maris, 56128 Pisa, Italy

Muscular dystrophies (MDs) make up a clinically and genetically heterogeneous group of skeletal muscle diseases with progressive muscle weakness and atrophy. The severity and distribution of affected muscles can vary dramatically in different forms and in single patients. This is partly due to a variety of genetic mutations that can cause different MDs. This Special Issue, entitled “Genetics of Muscular Dystrophies from Pathogenesis to Gene Therapy”, focuses on genetic backgrounds, genotype–phenotype correlations and potential modifiers in MDs, and novel therapeutic options driven by genetic characterization. Furthermore, it offers new insights into the field that may surpass the current limitations of therapies scattered across many forms. This Special Issue collects six original research articles, three review papers, and one case report study that aim to enhance our collective knowledge of muscular dystrophies and the genetic factors underlying these diseases.

Cotta and co-workers [1] described clinical and molecular data on a large cohort of Brazilian central core disease (CCD) patients, including a retrospective clinical analysis and molecular screening for RYR1 variants using next-generation sequencing (NGS). The authors analyzed 27 patients from 19 unrelated families, of which 4 had autosomal dominant (AD) and 2 had autosomal recessive (AR) inheritance. Biallelic *RYR1* variants were found in six families (two AR and four sporadic cases) out of the fourteen molecularly analyzed families (~43%), suggesting a frequency of AR inheritance that is higher than expected.

In a slightly different paper, Dosi and collaborators [2] adopted machine learning and unsupervised cluster analysis methods to untangle the clinical interpretation of gene variants in large muscular genes, using *RYR1* as an example. By analyzing the deep phenotype of 73 index cases harboring variants in *RYR1*, they were able to group 64/73 probands into four clusters with distinctive patterns of clinical and morphological findings. This new clustering approach is able to overcome the limits of current genotype–phenotype relationships in *RYR1*-related muscular disorders.

Viggiano and collaborators [3] report one of the largest single-center study on the spectrum of DMD variants observed in index cases with Duchenne (DMD) or Becker (BMD) muscular dystrophy. By annotating the precise genotype of 750 patients, the authors reported the prevalence of different types of mutations in patients with DMD and BMD according to their decade of birth, with relative percentages changing with time upon the introduction of NGS techniques. The authors documented the need for a timely and precise diagnosis in DMD and BDM in times of personalized therapies. Their study was perfectly aligned (?) with the review paper by Eser and Topaloğlu [4] who offered a current perspective of molecular therapies available for dystrophinopathies and muscular dystrophies in general, with a specific focus on exon skipping molecules. Some of these molecules have received conditional approval by health authorities and are being tested in numerous patients. 

Accuracy in molecular diagnosis is another major topic in the study conducted by Park and colleagues [5], who reported the first Korean Miyoshi muscular dystrophy type 1 case long misdiagnosed as BMD. The case report was an opportunity to review the multiple description of dysferlinopathy, the possibility of general manifestations, and the need for comprehensive diagnostic algorithms in muscle disorders. 

The role of genetic modifiers in the clinical manifestation of muscle diseases was investigated by Han and colleagues [6], who reviewed the intergenerational influence of the gender and phenotype of the transmitting parent on the occurrence of Korean myotonic dystrophy type 1, the most common autosomal dominant disorder caused by the CTG repeat expansion of the DMPK (DM1). Their data show that gender and the DM1 phenotype of the transmitting parent affect the CTG repeat size over subsequent generations. 

Using a different perspective, it was possible to discuss the role of modifiers in the hereditary inclusion body myopathies (HIBMs). Attri and colleagues [7] investigated the pathological mechanisms of the GNE-associated V727M mutation—the most prevalent ethnic founder mutation in the Asian HIBM group of patients—and showed that the mutation resulted in a deregulated lincRNA profile and a key target of these lincRNAs found in COL6A3. Further expression studies demonstrated that such deregulation could play a pivotal role in regulating many critical processes, including extracellular matrix organization, cell adhesion, and skeletal muscle development. Thus, studying this novel COL6A3-specific 13 gene signature provides new information which can help us to understand the molecular cause of HIBM and define a better diagnosis and effective therapeutic strategies for many muscular disorders.

Finally, three narrative reviews were able to define the spectrum of features of two different rare muscular dystrophies and one ultra-rare form. Chompoopong and Milone [8] have provided an extensive description of GDP-mannose pyrophosphorylase B (GMPPB)-related disorders. GMPPB disorders are inherited in an autosomal recessive manner and caused by mutations in either a homozygous or compound heterozygous states. The clinical spectrum of GMPPB-related disorders ranges from severe congenital muscular dystrophy (CMD) with brain and eye abnormalities to mild forms of limb–girdle muscular dystrophy (LGMD) to recurrent rhabdomyolysis without overt muscle weakness to features of neuromuscular junction impairment. On the other hand, Soontrapa and Liewluck [9] reported the spectrum of mono- and bi-allelic variants in anoctamin 5 (ANO5), including LGMDR12, Miyoshi distal myopathy type 3, pseudometabolic phenotype, and asymptomatic hyperCKemia. Murtazina and co-workers [10] described the first Russian patient with Native American congenital myopathy (NAM) due to pathogenic variants in the *STAC3* gene and hypothesized the possible contribution of muscle-specific isoforms on the clinical phenotype.

The articles included in this Special Issue cover a wide range of topics and provide innovative insights which can help to drive next-generation research in inherited muscular disorders. They also highlight that it is not only important for a complete and precise diagnosis [3,5] to propose timely personalized and pathogenic therapies [4,8], but also to consider potential genetic modifiers [6,7]. We anticipate that this Special Issue will help researchers dismiss concepts that are no longer applicable, such as the presence of homogeneous phenotypes [1,9,10] and the concepts of “one gene = one phenotype” to overcome the “single-dimension” paradigm traditionally used to describe genotype–phenotype relationships [2].
